# The effects of temperature stress and population origin on the thermal sensitivity of *Lymantria dispar* L. (Lepidoptera: Erebidae) larvae

**DOI:** 10.1038/s41598-022-26506-2

**Published:** 2022-12-17

**Authors:** Larisa Ilijin, Anja Grčić, Marija Mrdaković, Milena Vlahović, Dajana Todorović, Aleksandra Filipović, Dragana Matić, Vesna Perić Mataruga

**Affiliations:** Department of Insect Physiology and Biochemistry, Institute for Biological Research “Siniša Stanković”, National Institute of the Republic of Serbia, University of Belgrade, Despot Stefan Blvd. 142, 11060 Belgrade, Serbia

**Keywords:** Biochemistry, Ecology, Ecology, Environmental sciences

## Abstract

Increased environmental temperature is one of the most frequent stresses effecting metabolic rate in herbivorous insect species*.* Our goal was to compare the influence of increased environmental temperature and induced thermotolerance on the activity of midgut phosphatases and brain tissue hsp70 concentration in 5th instar *Lymantria dispar* larvae originating from an unpolluted and polluted forest. Induced thermotolerance (larval pre-treatment at high, sub-lethal temperature) increases the species ability to overcome the negative effects of thermal stress, therefore we monitored the effect of this regime in larvae originating from both forests. Thermal regimes in this experiment predominantly influenced the alkaline phosphatases activity and it was affected by temperature, population origin, and their combined effect. Total acid phosphatases activity was changed only by the joint effect of temperature and population origin. Brain hsp70 concentration was under a significant individual and joint effect of temperature and population. In both populations, brain tissue hsp70 concentration and alkaline phosphatases activity should be taken under consideration as a battery with biomarker potential for thermal stress in *L. dispar* larvae as a bioindicator species.

## Introduction

Human activities, primarily the burning of fossil fuels, have fundamentally raised the concentration of greenhouse gases in Earth’s atmosphere, warming the planet. Due to global warming, rising environmental temperature is one of the most frequent stresses confronting insects in nature, and thus their ability to resist it is a pivotal issue^1^. In an environment with raised temperature and CO_2_ concentration, plants increase their development, but with reduced nitrogen content in leaves^2^. In herbivorous insect species like *Lymantria dispar*, these interactions between environmental temperature and consumption of low protein level food can greatly impact growth, reproduction, and population dynamics^3^, especially in older, active feeding instars. They compensate for this protein deficiency by increasing their relative and total food consumption rates, meaning they eat faster and more^4^, imposing additional pressure on the herbivorous larval midgut. The influence of environmental temperature on metabolic rate is extreme, especially in herbivorous insects, where it can be indirect, through other factors, like the previously mentioned host plant composition, or direct through the impact of temperature on the physiology^1^. Due to their exothermic nature, virtually all aspects of insects` physiology and behavior are sensitive to environmental temperature. One of the most important direct effects of temperature is on the activity of enzymes, affecting the binding of the substrate to the enzyme and influencing the rate of enzymatically catalyzed reactions by shifting them towards those for which organisms possess sufficient energy in current conditions^5^.

Alkaline (ALP) and acid phosphatases (ACP) are located in insects` midgut, where they are included in the hydrolysis of different phosphomonoesters in alkaline and acid conditions, providing a pool of inorganic phosphates, and actively participate in food digestion^6^. ALP are predominant in insect larval midgut with high alkaline conditions^7^. The primary role of ALP is to provide phosphate ions from mononucleotides and ribonucleoproteins for various metabolic processes^8^. Besides this, they are included in insects` response reactions to stress or infection and the synthetic pathway of tyrosine, a precursor of biogenic amines (dopamine and octopamine) involved in the regulation of synthesis and release of insects` developmental hormones (see in^9^). The majority of ACP are soluble, mainly in the cytosol of midgut cells in Diptera and Lepidoptera, while a minority is membrane-bound in the gut lumen^10^. They participate in the final processes of metabolism of carbohydrates, phospholipids, and nucleotides, water resorption, signal transduction, and active membrane transport^11^. Lysosomes and components of the lysosomal compartment, like lysosomal acid phosphatase (lys ACP), which has a role in the hydrolysis of various macromolecules inside this compartment, appear to be very sensitive to different environmental stressors. Methods for tracking their activity are technically simple and low-cost, hence their increased implementation in monitoring the influence of anthropogenic pollution on aquatic biota^12^. Lys ACP has the potential for more usage as a reliable biomarker in forest insects.

Cellular response, primarily to heat stress but also other environmental stressors, involves in all organisms the synthesis of a small number of highly conserved proteins called heat-shock proteins (hsps). They are included in the transport, folding, and unfolding of proteins, regulation of insect diapause, act as molecular chaperones, etc. In many organisms, an hsp of 70 kDa (hsp70) is considered the major member of the hsp70 family^13^, and it consists of a constitutive and inducible form. At temperatures optimal for insect growth, they are located in the cell’s cytoplasm, whereas at increased temperatures, they can also be found in the nucleus^14^. Heat-shock proteins likely play an important role in insects` thermal adaptation and thermotolerance^15^.

Different insect species have developed thermotolerance as an ability to successfully overcome the effects of temperature changes^16,17^, and it can be an important factor in shaping the biogeographic distribution of insect species. Thermotolerance can be basic without prior acclimation and acquired, i.e. developed after pretreatment at an elevated but sublethal temperature. Tolerance to high temperatures can be induced by long or short term acclimatization of individual by rearing them at high, but sublethal temperatures for long or short period of time^18^. Conversely, induced thermotolerance is defined as increased thermotolerance after a pre-treatment at high, sub-lethal temperatures^19^. Individuals conditioned before the stress (induced thermotolerance) are probably more capable of surviving a heat shock that would be lethal to most unconditioned individuals.

Increases in temperature can enhance the toxicity of pollutants, or may alter their biotransformation to more bioactive metabolites, but also pollutants in the environment can modified the species thermal tolerance. This insect encounters various environmental stressors and has developed different adaptations to survive them. Such contact with different pollutants can increase the ability of an organism to cope with thermal stress (toxicant-induced thermal sensitivity), but it can be vice versa as well (climate-induced toxicant sensitivity)^20^.

Depending on locations, populations of the same species can respond differently to thermal stress^21^. *L. dispar* is a polyphagous lepidopteran species with a host range estimated at more than 500 plant species from 73 different families, widespread in the temperate climate zone. The northern boundary of *L. dispar* distribution will probably be shifted north by 500–700 km due to climate warming^22^.

In the current study, our goal was to compare the influence of increased sub-lethal environmental temperature with and without induced thermotolerance on midgut biochemical parameters such as the activity and isoform expression of ALP, tot ACP, lys ACP, and non-lys ACP, as well as brain tissue hsp70 concentration in 5th instar *L. dispar* larvae from two forests displaying varying levels of pollution (unpolluted and polluted). Combining selected parameters into an integrated biomarker response (IBR), we aimed to evaluate their sensitivity to thermal stress and to determine differences in responses between the two populations. Using larvae originating from two differently polluted natural populations (unpolluted vs polluted forest originating larvae), our goal was to reveal the influence of constant exposure to stressful environment and multigenerational exposure of *L. dispar* larvae to various environmental stressors in population originating from polluted forest, on their response to increased environmental temperature in comparison to larvae deriving from unpolluted forest. Using larvae originating from two differently polluted natural populations will indicate the importance of knowing the history of populations` previous exposure to other environmental stressors when monitoring forest ecosystems. Our results contribute to general knowledge about responses to thermal stress in phytophagous insects as a poll of potential biomarkers of changes in forest pollution, in order to preserve health and stability of forest ecosystems in future.

## Results

**Figure 1:**
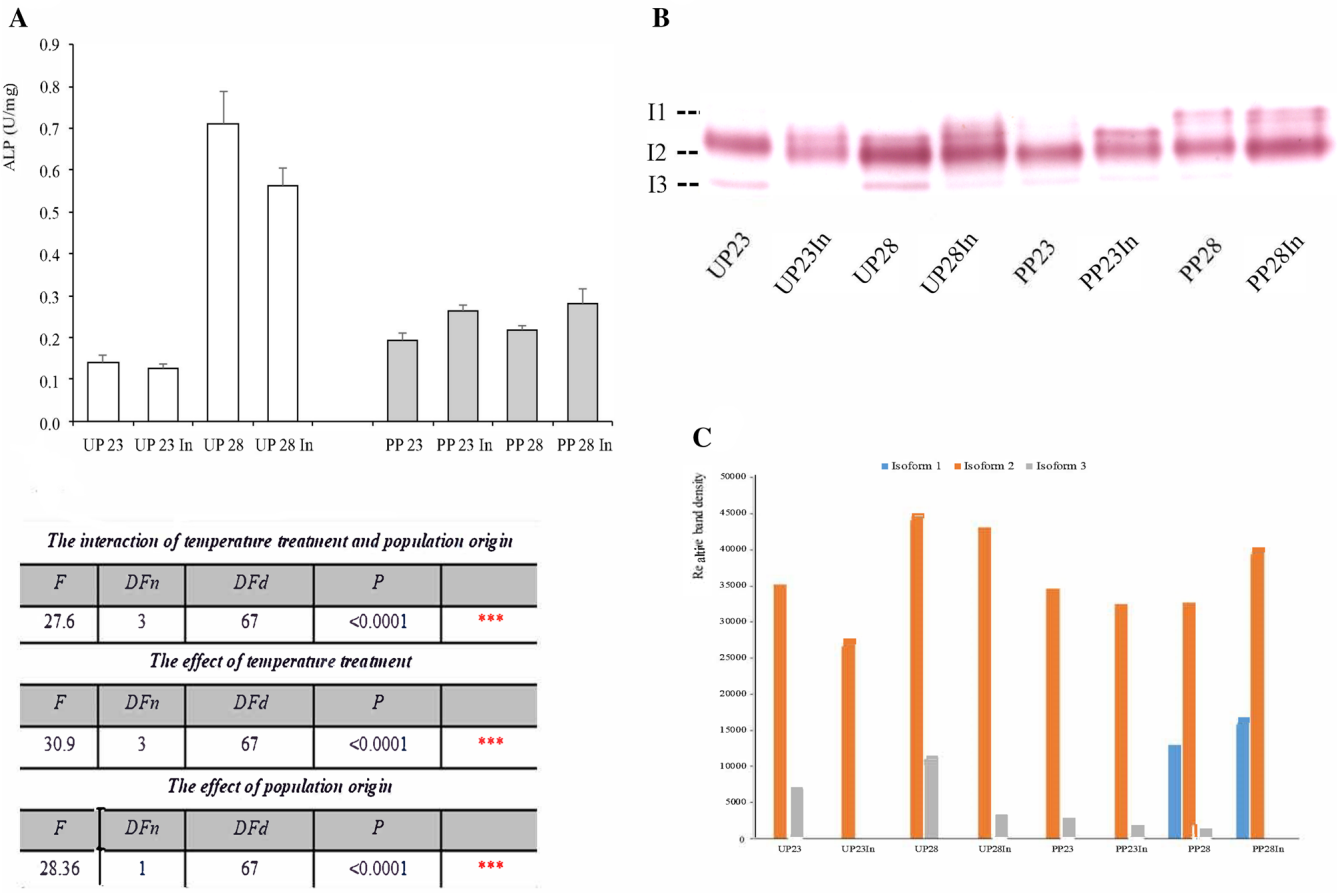
The specific activity of ALP in the midgut of 5th instar *Lymantria dispar* larvae exposed to different temperature treatments from unpolluted (UP) and polluted forest (PP) (**A**); UP 23 and PP23—larvae reared on 23 °C from hatching to sacrification; UP 23In and PP 23In-larvae reared on 23 °C from hatching until the first day of 4th instar, and then exposed to temperature of 28 °C for 24 h (induced thermotolerance). Afterwards they were returned to 23 °C until the third day of 5th larval instar; UP 28 and PP23—larvae reared on 23 °C from hatching until the first day of 5th larval instar, and then exposed to temperature of 28 °C for 72 h; UP 28 In and PP 28 In-larvae reared on 23 °C from hatching until the first day of 4th instar, and then exposed to temperature of 28 °C for 24 h (induced thermotolerance). Afterwards they were returned to 23 °C until the first day of 5th larval instar, and then exposed to temperature of 28 °C for 72 h. Error bars indicate the standard error of the mean (SEM) for (n = 8–11). Significance of the effects of the thermal treatments, population origin, and their interaction on the variance of ALP activity with thermal treatments as fixed factor (two-way ANOVA, *p* ≤ 0.05). Native PAGE gels stained for ALP with enzyme isoforms (**B**), and densitometric analysis of the bands using Image J program (**C**). Gel is cropped, and original gels are presented in Supplementary information, Figs. 1 and 2.

In the 5th instar larvae from the unpolluted forest, midgut ALP showed higher activity upon exposure to increased temperature with and without induced thermotolerance (Fig. 1a). In experimental groups from the polluted forest, induced thermotolerance was the factor that increase ALP activity at both temperatures. Using two-way ANOVA, we determined that the interaction of temperature treatments and population origin (unpolluted vs polluted forest; F_3, 67_ = 27.6, *p* < 0.0001) was extremely significant for changes in midgut ALP activity, together with the individual influence of increased temperature (F_3, 67_ = 30.9, *p* < 0.0001) and origin of the population of larvae (F_1, 67_ = 28.36, *p* < 0.0001) (Fig. 1a). In Fig. 1b, ALP isoforms detected on NATIVE gel electrophoresis were presented. Three isoforms were detected, but only one of them (isoform 2) was found in all experimental groups originating from unpolluted and polluted forest. Isoform 1 was present only in larvae from polluted forest exposed to 28 °C with increased band density in the group with induced thermotolerance. The second and dominant ALP isoform band intensities reflected the enzyme activity. Isoform 3 was not detected only in 2 groups, UP23In originating from unpolluted forest and PP28In from polluted forest, and there was a trend of lower band density of this isoform in larvae from polluted populations (Fig. 1c).

**Figure 2:**
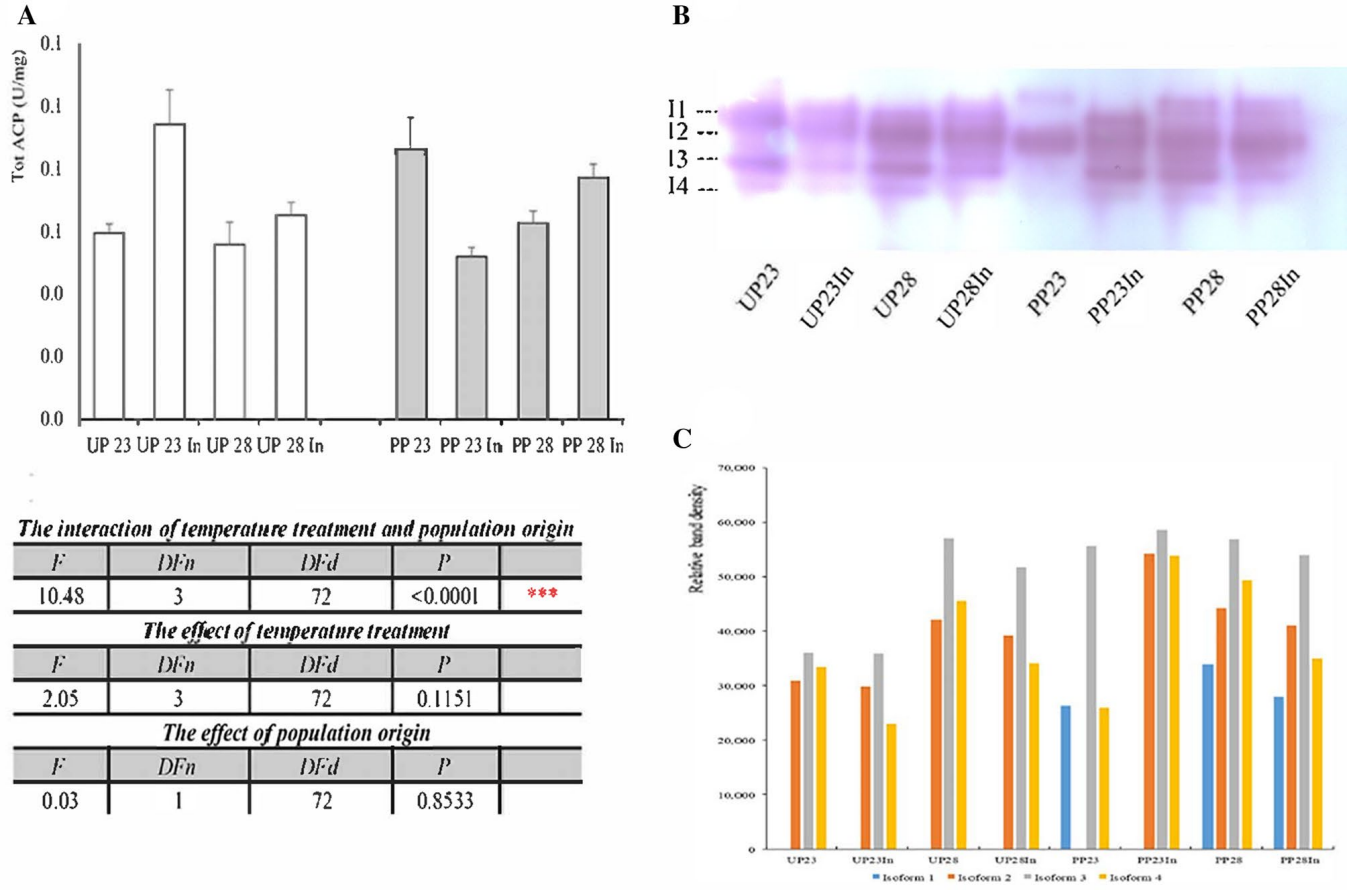
The specific activity of totACP in the midgut of 5th instar *Lymantria dispar* larvae exposed to different temperature treatments from unpolluted (UP groups) and polluted forest (PP groups) (**A**); All abbreviations are the same as in Fig. 1. Significance of the effects of the thermal treatments, population origin, and their interaction on the variance of totACP activity with thermal treatments as fixed factor (two-way ANOVA, *p* ≤ 0.05). TotACP isoforms detected on blot (**B**), and densitometric analysis of the bands using Image J program (**C**). Blot is cropped, and original blot is presented in Supplementary information, Fig. 3.

Changes in the activity of tot ACP in larvae from the unpolluted and polluted forest, depending on temperature treatment, are presented in Fig. 2a. In larvae originating from unpolluted forest, induced thermotolerance at both temperatures led to elevated tot ACP activity compared to non-induced groups. In contrast, in polluted forest`s larvae, all treatments decreased the enzyme activity in comparison to control. The interaction of temperature and population origin was extremely significant for tot ACP activity (two-way ANOVA, F_3, 72_ = 10.48, *p* < 0.0001), while their individual influence was not significant (Fig. 2a). Four isoforms of tot ACP were detected on the gel (Fig. 2b). Isoform 1 was present only in PP groups. Isoform 2 had a higher density in both populations and all treatments in comparison to controls. High band density of isoform 3 was present in all experimental groups, while induced thermotolerance and increased temperature led to high band density of isoform 4 in both populations (Fig. 2c). Induced thermotolerance affected the activity of tot ACP in caterpillars originating from the unpolluted forest, while in caterpillars from the polluted habitats, it was decreased in all treatments, and a new isoform band was detected on NATIVE gels.

**Figure 3:**
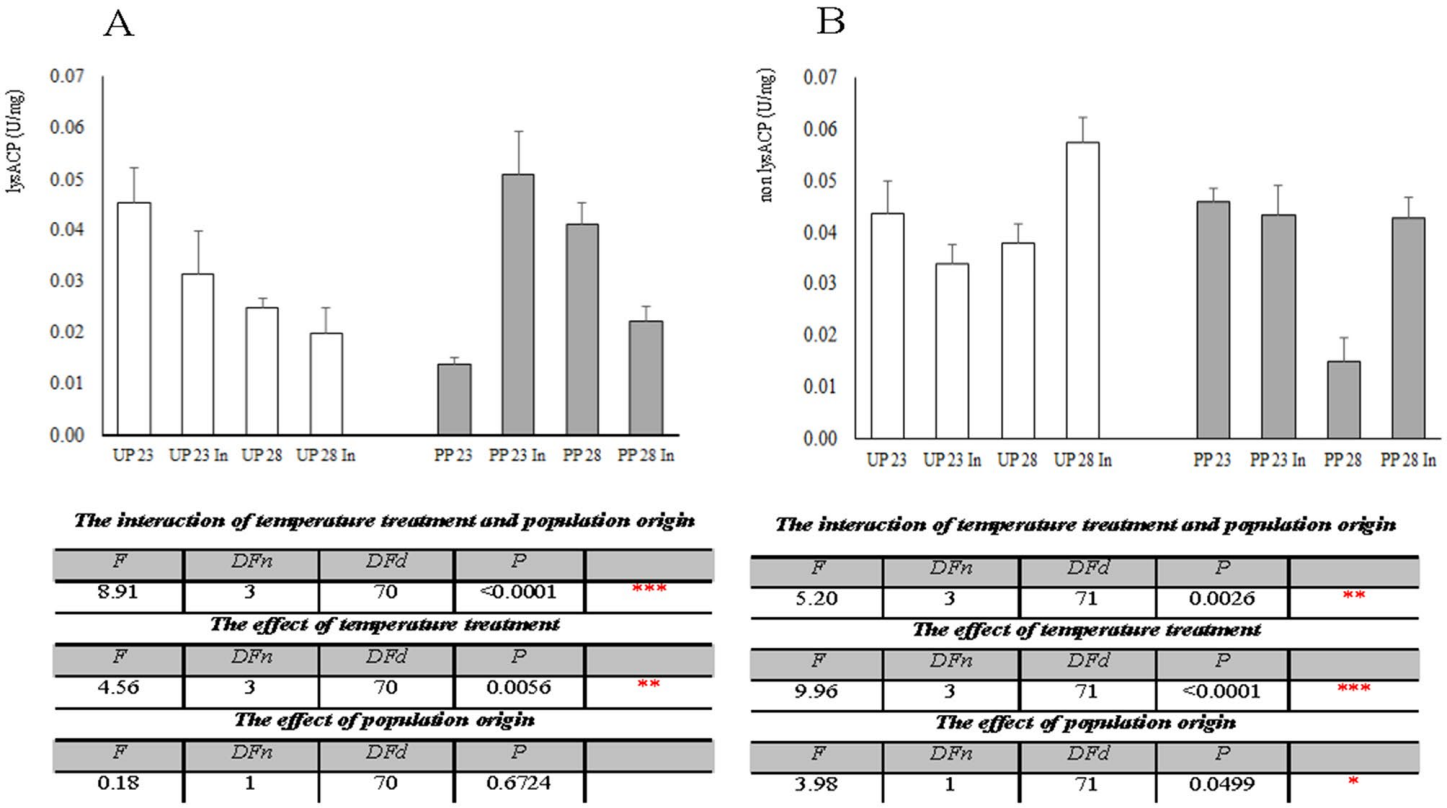
The specific activity of lysACP (**A**) and nonlysACP (**B**) in the midgut of 5th instar *Lymantria dispar* larvae exposed to different temperature treatments from unpolluted (UP groups) and polluted forest (PP groups); All abbreviations are the same as in Fig. 1. Significance of the effects of the thermal treatments, population origin, and their interaction on the variance of lysACP and nonlysACP activity with thermal treatments as fixed factor (two-way ANOVA, *p* ≤ 0.05).

Changes in lys and non-lys ACP activity in all experimental groups are presented in Fig. 3a and b, respectively. In larvae originating from the unpolluted forest, the activity of this enzyme was decreased at all temperature treatments in comparison to the control group, while the situation was opposite in polluted forest`s groups, where its activity was increased at all thermal regimes (Fig. 3a). Two-way ANOVA analysis (Fig. 3a) revealed that the interaction of temperature treatment and population origin was extremely significant (F_3, 70_ = 8.91, *p* < 0.0001), whereas the effect of temperature treatment was considered very significant (F_3, 70_ = 4.56, *p* = 0.0056). The activity of non-lys ACP is presented in Fig. 3b. In larvae from the unpolluted forest, the activity of this enzyme was increased only in the UP28In group of larvae originating from unpolluted forest, while this enzyme’s activity did not change in treatments with larvae originating from the polluted forest, except for the decreased activity in the PP28 group from polluted forest. The interaction between temperature treatment and population origin was found to be very significant (two-way ANOVA, F_3, 71_ = 5.20, *p* = 0.0026), and the effect of temperature treatment was extremely significant (two-way ANOVA, F_3, 71_ = 9.96, *p* < 0.0001). The influence of population origin was considered significant (two-way ANOVA, F_3, 71_ = 3.98, *p* = 0.0499).

**Figure 4:**
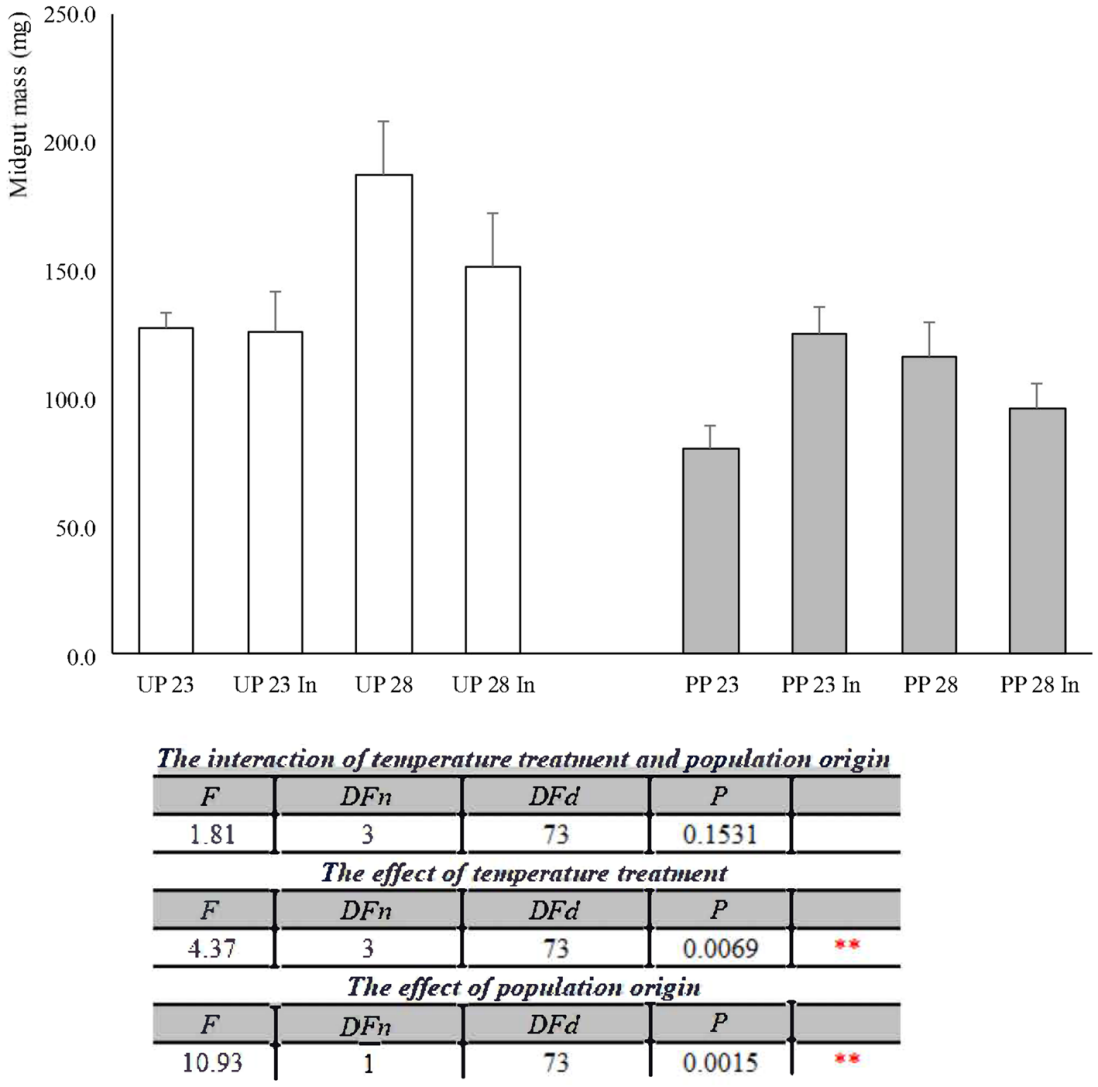
The midgut mass of 5th instar *Lymantria dispar* larvae exposed to different temperature treatments from unpolluted (UP groups) and polluted forest (PP groups). All abbreviations are the same as in Fig. 1. Significance of the effects of the thermal treatments, population origin, and their interaction on the variance of larval midgut mass with thermal treatments as fixed factor (two-way ANOVA, *p* ≤ 0.05).

Midgut mass in the 5th instar *L. dispar* larvae from all experimental groups is presented in Fig. 4. In the group from the unpolluted forest, exposure to elevated temperature with and without induced thermotolerance led to higher larval midgut mass. In all groups of larvae deriving from polluted forest, larval midgut mass was increased in comparison to the control group. Two-way ANOVA analysis revealed that the interaction of temperature treatment and population origin was not significant, while the effect of temperature treatment was very significant (F_3, 73_ = 4.37, *p* = 0.0069), as well as population origin (F_1, 73_ = 10.93, *p* = 0.0015).

**Figure 5:**
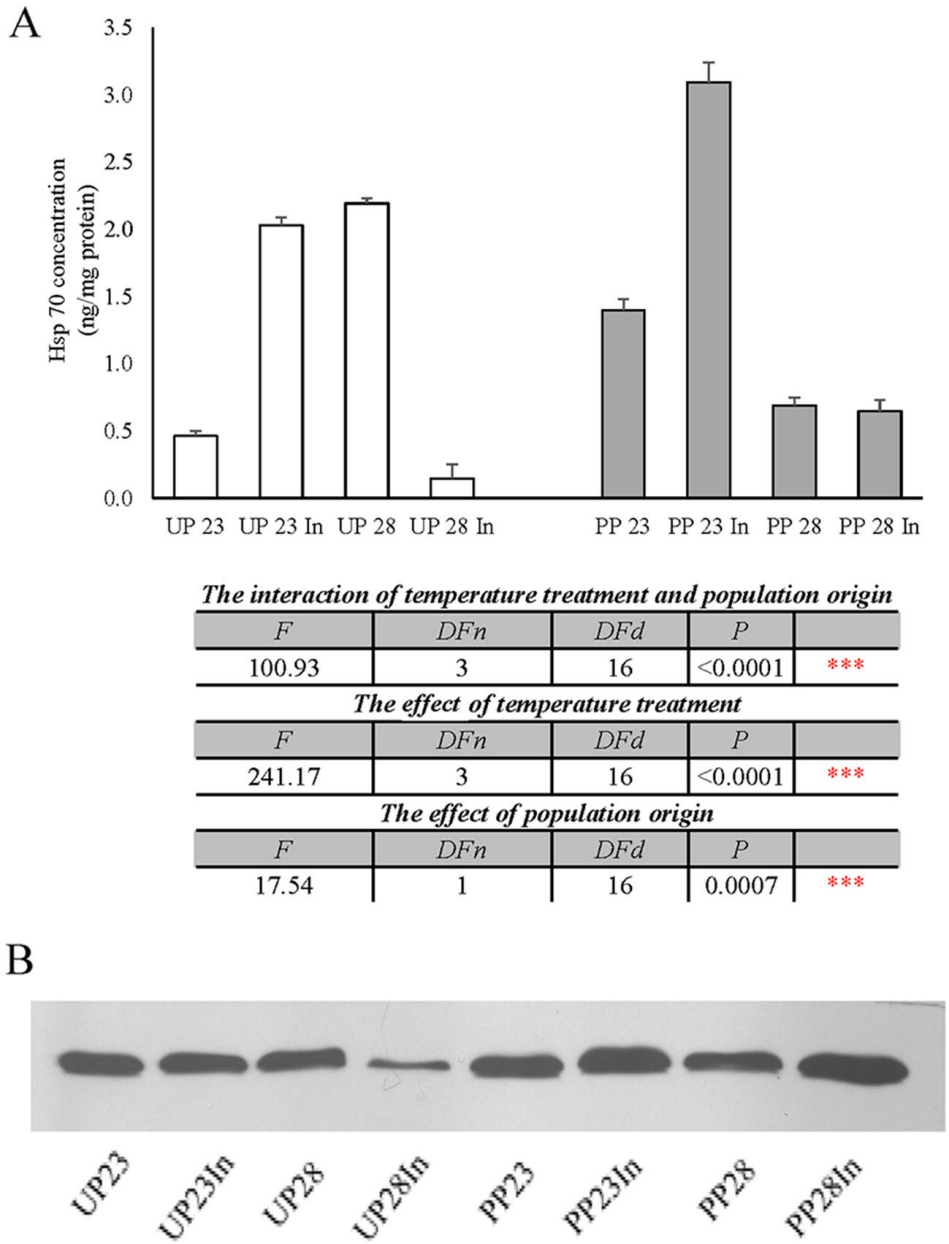
The total brain tissue hsp70 concentration quantified by indirect ELISA in 5th instar *Lymantria dispar* larvae exposed to different temperature treatments from unpolluted (UP groups) and polluted forest (PP groups) (**A**). Data expressed as mean ± standard error, ng/mg of proteins for (n = 15 larvae in each experimental group); All abbreviations are the same as in Fig. 1. Significance of the effects of the thermal treatments, population origin, and their interaction on the variance of larval brain hsp70 concentration, with thermal treatments as fixed factor (two-way ANOVA, *p* ≤ 0.05). Western blot of heat shock protein 70 (hsp70) from brain tissue (**B**). Western blot film is cropped, and original film is presented in Supplementary information, Fig. 4.

Figure 5 presents changes in the brain tissue level of hsp70 after exposure to different thermal regimes in larvae from both populations. In groups originating from the unpolluted forest, hsp70 concentration was increased in UP23In and UP28 groups from unpolluted forest (Fig. 5a). In larvae originating from the polluted forest, the level of this enzyme was increased only in the PP23In group from polluted forest, while in 28 °C thermal regimes, it was decreased compared to control (Fig. 5a). Two-way ANOVA analysis (Fig. 5a) revealed that the interaction of temperature and population origin, and individual influences of temperature treatment and population origin were extremely significant (F_3, 16_ = 100.93, *p* < 0.0001; F_3, 16_ = 241.17, *p* < 0.0001; F_1, 16_ = 17.56, *p* = 0.0007, respectively).

**Figure 6:**
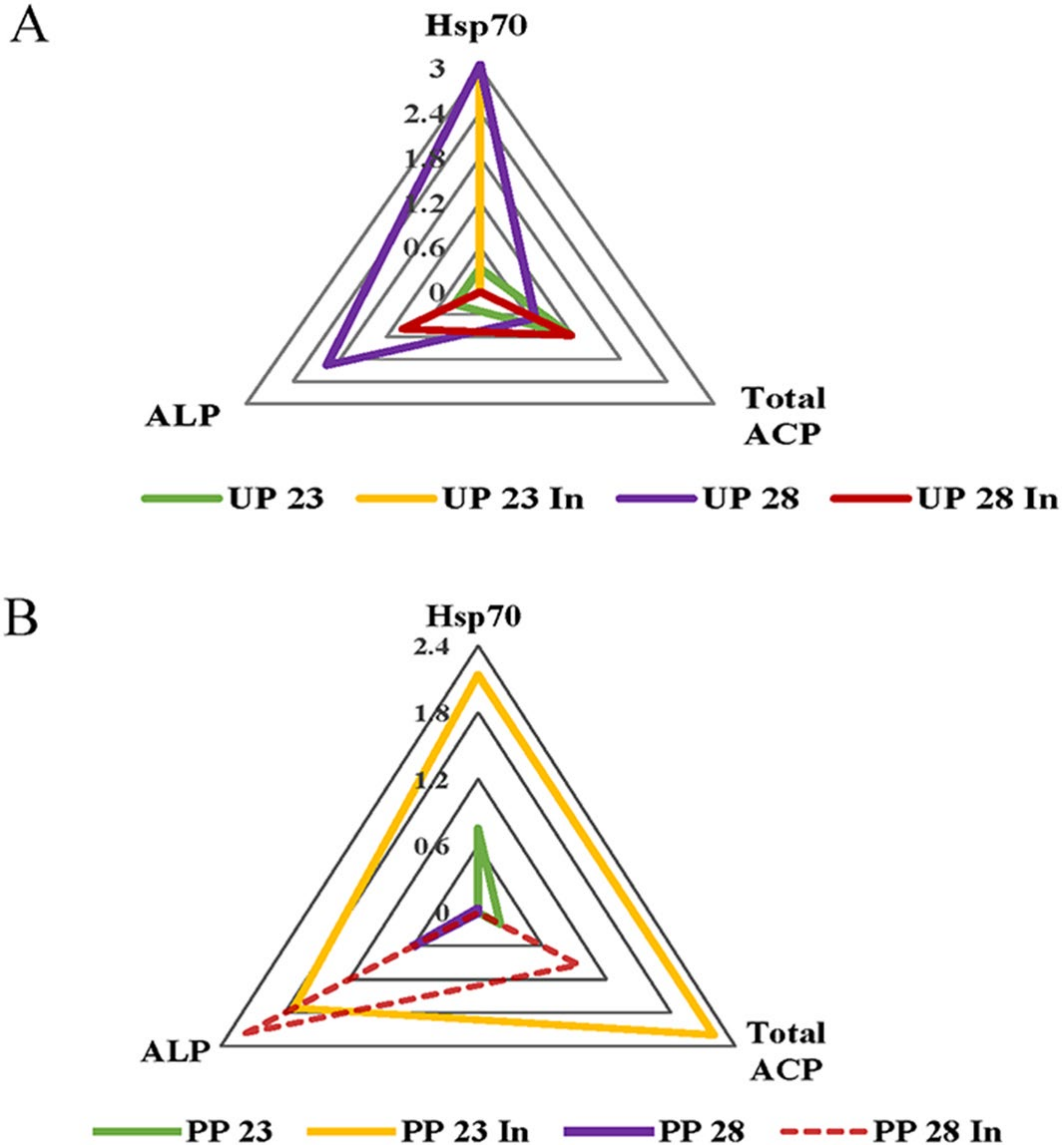
IBR index calculated for midgut alkaline phosphatases (ALP), midgut total acid phosphatases (total ACP) and brain tissue hsp70 for 5th instar *Lymantria dispar* larvae exposed to different temperature treatments from unpolluted forest (**A**) and polluted forest (**B**). All abbreviations are the same as in Fig. 1.

The IBR index (Table 1) showed that in the groups of larvae originating from the unpolluted forest, the highest score was registered for brain hsp70 concentration and ALP activity in the UP28 group and hsp70 level in the UP23In group, both originating from unpolluted forest (Fig. 6a). In larvae from the polluted forest, the highest IBR index was detected for brain tissue hsp70 level and tot ACP activity in the PP23In group and ALP activity in the PP28In group (Fig. 6b).

**Table 1:** IBR index values for midgut alkaline phosphatases (ALP), midgut total acid phosphatases (total ACP) and brain tissue hsp70 for 5th instar *Lymantria dispar* larvae exposed to different temperature treatments.

	Experimental treatments	IBR index
*L. dispar* larvae originated from unpolluted forests (UP)	UP23	0.31
	UP23In	0
	UP28	3.33
	UP28In	0.12
*L. dispar* larvae originated from polluted forests (PP)	PP23	0.061
	PP23In	3.201
	PP28	0.009
	PP28In	0.689

## Discussion

Thermal sensitivity can be assessed at a number of levels: from animal activity through energy status and, finally, to mortality. For the majority of forest insect species, the development of egg, larva, and pupa shortens at higher temperature conditions. A moderate increase in temperature is linked with an increased CO_2_ concentration and, consequently, the decrease of nitrogen level in foliage and increased synthesis of plant secondary metabolites^2^. For foliophages, this increasing temperature causes a reduction in food quality, resulting in increased feeding and acceleration of insect metabolism^23^. For insects, resting metabolic rate, i.e., fundamental biochemical processes necessary for survival, requires approximately up to 50% of individual energy disposal^24^. Periods of increased metabolic rate, induced by elevated environmental temperature, are accompanied by higher oxygen consumption and the generation of free radicals^25^. This could be lethal for organisms due to increased energy requirements (extended requirement of phosphate ions) necessary for activating antioxidative defense and other physiological stress-protective mechanisms^26–28^. The central role of phosphatases is the hydrolysis of phosphomonoester and transphosphorylation^11^.

Insects` ALP are located mainly in the midgut, and they are actively involved in several phases of food digestion, like absorption of metabolites and transport processes^29^. According to Miao^30^, the lowest activity of this enzyme was detected in *Bombyx mori* during larval molting. After that, the activity increased, and the highest activity was seen at the 5th instar just before full active feeding, decreasing again at mature stages. Also, the ALP activity in larval midgut was dependent on the qualities of host plant leaves and environmental temperature. The highest activity was detected in larvae fed with young leaves and reared under optimal environmental temperature. Increased temperature induces a rise in ALP activity^30^. In *Drosophila virilis* larvae, prolonged exposure to high temperature produced an increase in ALP activity^31^. In our experiment, in *L. dispar* 5th instar larvae originating from the unpolluted forest (UP groups), an increase in ALP activity was observed only in groups exposed to increased environmental temperature with and without induced thermotolerance (Fig. 1). This instar is in an active feeding state and consumes large amounts of food, obviously due to increased rearing temperature, since this kind of response is not present in groups reared under normal temperature, regardless of induced thermotolerance. Ilijin et al.^28^ recorded a rise in the relative growth rate of 5th instar *L. dispar* larvae upon exposure to 28 °C. Increased metabolic activity causes the increased ALP activity in these unpolluted forest`s groups, probably as an adaptive mechanism, as it is known that this flexible activity of enzymes runs more effectively at higher temperatures. Our previous results^28^ indicate an increased level of ROS in 5th instar *L. dispar* larvae originating from an unpolluted forest and exposed to increased environmental temperature, since the activity of antioxidative (AOX) enzymes superoxide dismutase and catalase was increased in midgut and hemolymph. This activation of AOS requires the generation of extra energy, i.e., phosphate ions, and therefore increased activity of ALP as well. In experimental groups with larvae originating from the polluted forest (PP groups), ALP activity was not sensitive to elevated environmental temperature (Fig. 1) due to their exposure to various environmental stressors through several generations, which probably improved their ability to overcome the adverse effects of the hotter environment. In groups with induced thermotolerance originating from polluted forest, ALP activity was elevated compared to those without induced thermotolerance (Fig. 1). Short-term induced thermotolerance secures the protection of organisms at very high temperatures, so we think that in these experimental groups, 28 °C represents a critical threshold for ALP activation.

Among the three ALP isoforms detected on NATIVE gels (Fig. 1), isoform 1 is present in all experimental groups, and band density changes track changes in ALP activity. Isoform 2 is present at almost all treatments. Still, its band density was lower and responded to temperature in a population-dependent manner: in unpolluted forest`s larvae, band density was low or absent after induced thermotolerance and increased after elevated temperature exposure. On the other hand, band density was very low in all polluted forest`s experimental groups. This isoform’s sensitivity to thermal stress depends on insects` thermal adaptation, obtained by induced thermotolerance or their previous contact with other pollutants (toxicant-induced climate change sensitivity). Isoform 3 was only present at thermal treatments in larvae originating from the polluted forest. The obtained data led us to the conclusion that, besides one constantly active ALP isoform, increased tolerance to thermal stress due to induced thermotolerance or exposure to other pollutants for several generations is associated with the expression of other isoforms of this enzyme, which are expressed at low levels or suppressed at optimal temperature conditions for the 5th instar *L. dispar* larvae. Dynamic modulation in the expression of a particular isoform can be more beneficial for the maintaining the homeostasis in organism, than changes in its total activity, in the state of stress exerted by environmental agents. In the brain tissue of fifth-instar larvae we detected different numbers and intensities of isoform bands, which could indicate different sensitivity of analyzed enzyme to applied thermal regimes and population origin (unpolluted vs polluted forest), as well as higher enzyme plasticity expressed in response to them.

Acid phosphatases are the other group of phosphatases, active in acid conditions mainly in the cytosol of midgut cells of Lepidoptera^11^ but also in Malpighian tubules, muscles, and nerve ganglia^32^. They catalyze the hydrolysis of a variety of phosphate monoesters and phosphoproteins. This increased production of phosphate ions is important for the high energy demanding stress response mechanism in insects. Lys ACP is included in the hydrolysis of various macromolecules inside the lysosomal compartment, and the lysosomal enzyme release assay is considered a biomarker of exposure to toxicants^12^. Vlahović et al.^33^ detected irreversible inhibition of lys ACP upon chronic exposure of *L. dispar* larvae to cadmium, while benzo(a)pyrene-induced chronic toxicity had a slight effect on the activity lys ACP^34^. Thermal stress disturbed the normal redox cycle in *L. dispar* larvae^28^, while induced oxidative stress decreased acid phosphatase activity in *B. mori*^35^. In our experiment in *L. dispar* 5th instar larvae, the activity of midgut tot ACP was increased only in the UP23 group with induced thermotolerance, originating from unpolluted forest, which could be a response to short—term exposure to high temperature. In groups deriving from polluted forest, its activity was decreased at all thermal treatments in comparison to control group of larve from polluted forest (Fig. 2). Comparing the control group from unpolluted forest to the one from polluted forest, it is visible that the activity in the second is higher. This is most probably the results of the higher pollution presure present in polluted forest from Lipovica, due to proximity of heavy trafic. This increased level of various pollutants causes the activation of high energy demanding stress response mechanism in insects, and increased level of ACP for catalyzation of the hydrolysis of a variety of phosphate monoesters and phosphoproteins, and increased production of phosphate ions as an source of necessary energy. We can presume that both alkaline and acid phosphatases are included in the generation of energy necessary for stress protective mechanisms.

On native gels, 3 isoforms of tot ACP were detected (Fig. 2). Isoform 1 was found only in groups originating from the polluted forest, and band density was high in groups exposed to elevated temperature, indicating this tot ACP isoform is activated in response to thermal stress only in larvae that had previous contact with other pollutants. Isoform 2 was present in almost all experimental groups, with higher band density in groups with toxicant-induced climate change sensitivity. The third isoform was detected at all treatments. Protein band density was increased in larvae originating from the unpolluted forest exposed to 28 °C with and without induced thermotolerance and in all experimental groups compared to larvae from the polluted forest. Isoform 4 was seen in all groups, with higher expression in polluted forest groups. The general conclusion is that the expression of tot ACP isoforms upon thermal stress also depends on population origin. Three isoforms were present in groups originating from the unpolluted forest. Their expression was the highest at 28 °C in comparison to the control group and those with induced thermotolerance, which made this enzyme slightly less sensitive to elevated temperature. In groups originating from the polluted forest, a completely new isoform was active in both control and treated groups as a response to increased levels of pollutants in general in this forest. The lysosomal component of this enzyme showed suppressed activity in all unpolluted forest groups, whereas its activity in all groups from polluted forest was increased compared to the control. Non-lys ACP activity was decreased in unpolluted forest deriving groups, except in the UP28In group, where it was increased. In larvae originating from the polluted forest, non-lys ACP activity was at the control level except in larvae exposed to 28 ℃, where this enzyme was suppressed (Fig. 3).

On the basis of our results, we presume that thermal regimes performed in this experiment predominantly influenced the activity of *L. dispar* midgut ALP compared to ACP, most probably due to alkaline conditions in the larval midgut. Also, temperature and population origin and their joint effect significantly influenced the activity of ALP. By contrast, tot ACP activity was affected only by the joint effect of temperature and population origin. Lysosomal and non-lysosomal ACP were significantly influenced by the joint effect of temperature and population origin. Lys ACP and non-lys ACP were under a significant effect of temperature and population origin and individual effect of temperature as well, and the non-lys component was further affected by population origin. These data indicate that both factors, thermal regimes and population origin, influence the activity of analyzed phosphatases, meaning that previous contact of larvae with other stressors should not be omitted when estimating their response to the effects of thermal stress in *L. dispar* larvae. Elevated ambiental temperature can strongly enhance the accumulation of pollutants in environment and in insects tissues due to increased locomotion and consequently increased food consumption, but also because of disturbed dynamics of pollutant penetration across biological membranes^36,37^. These changes modifies consequently insect physiological processes, their stress protective mechanisms (like analyzed phosphatases), and impact insects` life history performances, their number and distribution in forests.

Midgut larval mass was higher in larvae from the unpolluted forest exposed to increased temperature with and without induced thermotolerance, while in those from the polluted forest, all thermal treatments led to an increase in midgut mass (Fig. 4). These changes were only individually induced by elevated temperature or population origin, while their joint effect did not significantly affect midgut mass.

After exposure to temperatures higher than normal temperatures required for growth cells, organisms produce heat-shock proteins (hsps)^38^, with hsp70 being the most commonly described in the insect heat response. Increased hsp70 transcription and translation levels occur as a response to various environmental stressors. In *L. dispar* larvae, a fluctuating response to ingested benzo(a)pyrene and fluoranthene^39^ was detected in the brain tissue hsp70 concentration^40^, and midgut hsp70 expression was elevated in larvae originating from a polluted forest^34^.

In this experiment, in both populations, hsp70 synthesis was induced in larvae reared at optimal temperature upon exposure to induced thermotolerance. In larvae originating from the unpolluted forest, hsp70 synthesis was initiated at increased temperature. When thermotolerance was induced, however, the concentration of this protein was very low, probably due to the ʺimmunizationʺ of larvae to this stressor. Larvae originating from polluted forest from PP 28 and PP28Ind groups showed decreased levels of hsp70, which could be a consequence of this population's previous multigenerational contact with other pollutants and generated tolerance to increased temperatures. Perić et al.^41^ detected an increased level of hsp 70 expression in *L. dispar* larvae brain tissue originating from polluted forest upon exposure to cadmium and combined cadmium with increased temperature, but increased temperature did not have an up regulated effects on hsp 70. Since the induction of hsp 70 synthesis is energetically expensive process in insects, it is also possible that activation of other, cheeper stress protective mechanism might mitigate the damage coused by pollution in *L. dispar* larvae originating from polluted forest. Hsp70 concentration in this experiment was under a significant effect of temperature and population origin, but also the individual effects of temperature and population origin. The monoclonal anti-heat shock protein-70 used in our Western blot analysis localizes both the constitutive (hsp73) and inducible (hsp72) forms, therefore further analysis is needed to determine if a switch off in expression of the constitutive and/or inducible hsp70 form is present, and if it is dependent on thermal regime or population origin, i.e. previous multigenerational contact with various environmental stressors.

Using *L. dispar* larvae as a bioindicator organism in thermal stress assessment, the parameters we have analyzed reveal population adaptation differences and changes in enzyme activity and stress protein concentrations upon induced thermotolerance and increased rearing temperature. Therefore, it was important to determine which combinations of parameters could be used as potential biomarkers in thermal stress biomonitoring. Using IBR analysis, the highest score was registered for brain tissue hsp70 concentration and ALP activity in the UP28 group and brain tissue hsp70 level in UP23Ind from unpolluted forest. In larvae of polluted origin, the highest IBR score was observed for brain tissue hsp70 level and tot ACP activity in the PP23In group and ALP activity in the PP28In group, both groups of larvae originating from polluted forest. Since brain tissue hsp70 level and ALP activity had the highest scores upon thermal stress in both populations, these parameters should be taken under consideration as a potential biomarker battery for the indication of thermal stress.

## Conclusion

Thermal regimes in this experiment predominantly influenced the activity of *L. dispar* midgut ALP, most probably due to alkaline conditions in the larval midgut. Differences in ALP sensitivity to increased temperature and induced thermotolerance were evidently affected by temperature, population origin, and their combined effect. On the other hand, tot ACP activity was changed only by the joint effect of temperature and population origin. Lys ACP and non-lys ACP were significantly affected by temperature and population origin, and also individually by temperature, whereas the non-lys component was influenced by population origin as well. Brain tissue hsp70 concentration was under a significant effect of temperature and population origin and the individual effect of temperature and population origin. In the IBR analysis, the highest IBR score in both populations was registered for brain tissue hsp70 concentration and ALP activity; therefore, these parameters should be taken under consideration as a battery with biomarker potential for thermal stress in *L. dispar* larvae as a bioindicator species.

## Material and methods

In the autumn (November), *L. dispar* egg masses were collected at two sites: unpolluted and polluted forest. The first was a mixed oak forest at Kosmaj Mountain, 40 km south-east of Belgrade (coordinates 44°27′56″N 20°33′56″E). These woods are regarded as unpolluted because they are far from direct pollution and are part of the system of protected green areas around Belgrade, where the construction of industrial facilities and traffic infrastructure with potential negative effects on the environment is prohibited by legal regulations. The second site was Lipovica Forest (coordinates 44°38′11″N 20°24′12″E), with mixed *Quercus frainetto* and *Quercus cerris* trees, considered a polluted forest since it is located along the border of State Road 22, one of the most frequently used IB-class roads in Serbia.

**Figure 7:**
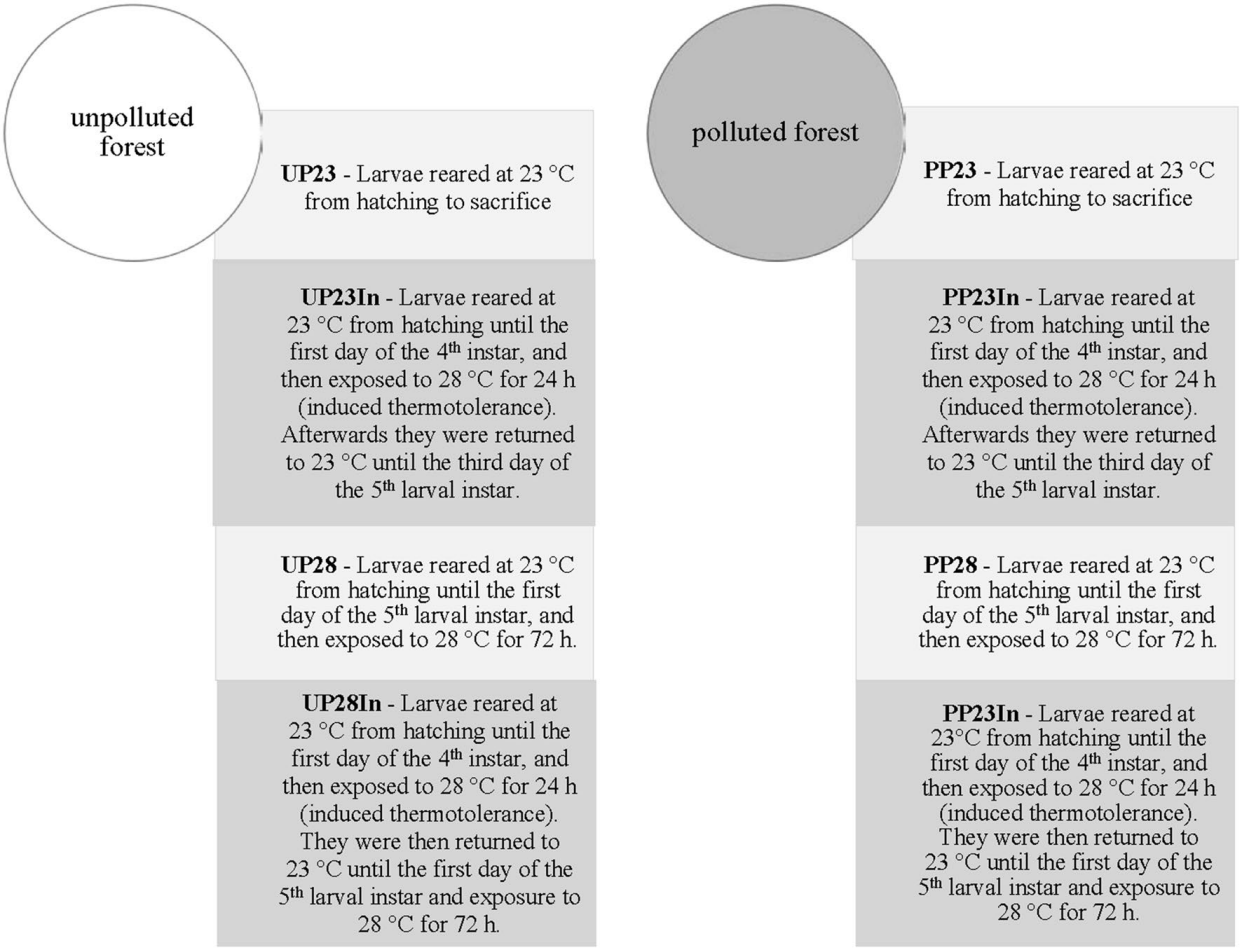
A schematic figure of the experimental treatments.

Collected egg masses were kept in a refrigerator at 4 °C until spring (March) when 200 eggs for each experimental group were set for hatching. After hatching in transparent Petri dishes (V = 200 mL), 10 first instar larvae were transferred and reared together at 23 °C with a 12:12 h light: dark photoperiod and relative humidity of 60%, until the third larval instar. Then, five 3rd instar larvae were reared together in the same Petri dish. After molting into the 4th instar, each larva was kept individually until the third day of the 5th instar, when they were sacrificed. Larvae were fed on an artificial diet designed for *L. dispar*^42^, and food was replaced every 48 h. Each experimental group contained between 50 and 60 larvae (Fig. 7).

The optimal temperature for *L. dispar* larval development is 23 °C, and the control group was reared at this temperature. The highest summer temperature (2007–2010) measured in Serbian Quercus forests at a similar elevation was 28.4 °C, and the lowest 19.6 °C, while the average summer temperature was 26.3 °C^43^. Thus, we established variable temperature regimens that included brief (24 h) and daily (72 h) exposures to 28 °C. The control group of larvae were reared through the whole experiment on optimal 23 °C. Results of Huey et al.^44^ indicate that short term (daily) exposure to higher temperatures during development can increase both optimal temperature and maximal growth rate at the optimum, an example of beneficial thermal acclimation. In our previous research we found that induced thermotolerance modifies the activity of detoxifying enzymes in larvae originating from the polluted forest. We exposed *L. dispar* larvae in several experimental groups to that regime at 4th larval instar, with intention of analyze the effects of induce thermotolerance on observed parameters (ALP, ACP, hsp 70) in 5th instar larvae reared on optimal or elevated temperature^28^.

At sacrifice on the third day of the 5th instar, the caterpillar midguts were dissected out on ice (n = 8–11 larval midguts per group for each enzyme assay). Midgut from single larvae was weighed and homogenized in insect physiological saline, as insect fluids have buffer values similar to vertebrates^45^. Homogenization was performed in ice-cold 0.15 M NaCl (final tissue concentration was 100 mg/mL in each sample), for 3 intervals of 10 s with a 15 s pause between them, at 5000 rpm, using Ultra Turrax homogenizer (IKA-Werke, Staufen, Germany). The homogenates were centrifuged for 10 min at 10,000 g at 4 ℃, and supernatants were used for enzyme assays and NATIVE gel electrophoresis. This protocol ensured that supernatants would contain cytosol and lysosomes.

On the third day of the 5th instar, larval brain tissues were dissected out on ice and weighed. Pooled brain tissue (n = 30 brain tissues per experimental group) was diluted with 0.9% NaCl (1:9/w:V) and homogenized on ice at 5000 rpm during three 10 s intervals, separated by 15 s pauses (MHX/E Xenox homogenizer, Germany). Homogenates were centrifuged at 25,000 g for 10 min at 4 °C in an Eppendorf 5417R centrifuge (Germany). The supernatants were used for Western blotting and indirect non-competitive enzyme-linked immunosorbent assay (ELISA). Protein concentrations samples were determined using BSA as the standard^46^.

A modified method by Nemec and Socha^47^ was used to determine the activity of ALP. The reaction mixture contained 0.1 M Tris HCl buffer pH 8.6, 5 mM MgCl_2_, midgut homogenate, and 5 mM *p*-nitrophenyl phosphate. During 30 min of incubation time at 30 ℃, the hydrolytic release of *p*-nitrophenol from *p*-nitrophenyl phosphate (pNPP) occurred under alkaline conditions.

The reaction was stopped with 0.5 M NaOH, and the absorbance of p-nitrophenol was measured at 405 nm. Blank and non-catalytic probes were included. One unit of enzyme activity was defined as the amount of enzyme that released 1 mmol of p-nitrophenol per minute under the assay conditions.

The same modified method of Nemec and Socha^47^ was employed to determine ACP activity, but under acidic conditions (0.1 M citrate buffer pH 5.6 was found optimal for *L. dispar* ACP), with a prolonged incubation time of 60 min. One unit of enzyme activity was defined as the amount of enzyme that released 1 μmol of *p*-nitrophenol per minute per mg of total protein. Total ACP activity determined in the midgut samples came from lysosomal ACP that ended up in the cytosol and non-lysosomal ACP, typically localized in the cytosol.

Lysosomal ACP were detected indirectly^48^, under the same conditions, in a mixture containing the specific enzyme inhibitor NaF (50 mM). The absorbance determined at 405 nm is proportional to the activity of the non-lysosomal fraction of total ACP. The activity of the lysosomal fraction was obtained by subtracting not inhibited non-lysosomal acid phosphatases from the total phosphatase activity. Specific activities of ACP are given in mU per mg of total protein.

A modified method by Allen et al.^49^ was used to detect ALP isoforms after native PAGE. Using 12% polyacrylamide gel, 10 μg protein aliquots per well were separated at 100 V and 4 ℃. The ALP isoform activity was visualized by soaking the gel in an incubation mixture consisting of 0.13% α-naphthyl phosphate, 100 mM Tris–HCl buffer (pH 8.6), and 0.1% Fast Blue B. The gels were incubated at room temperature until bands appeared.

For ACP phosphatase detection, the same method of Allen et al.^49^ was also modified. After electrophoresis, the gel was washed with deionized water and equilibrated in 100 mM acetate buffer (pH 5.2) at 30 ℃. The nitrocellulose membrane was pre-soaked in 0.13% α-naphthyl phosphate dissolved in the same acetate buffer for 50 min at room temperature. The gel was covered with the membrane and incubated in a moist chamber for 60 min at 30 ℃. The membrane was soaked in 0.3% Fast Blue B stain dissolved in acetate buffer until bands became visible.

Gels were scanned with a CanoScan LiDE 120 (Japan). The intensities of enzyme bands in the regions of ALP and ACP activities were analyzed using the ImageJ 1.42q software (U. S. National Institutes of Health, Bethesda, Maryland, USA).

An indirect non-competitive ELISA was used to quantify the concentration of hsp70 in *L. dispar* brain tissue. Samples were diluted with carbonate-bicarbonate buffer (pH 9.6) and coated on a microplate (15 μg of tissue/well) (Multiwell immunoplate, NAXISORP, Thermo Scientific, Denmark) overnight at 4 °C, in the dark. The indirect non-competitive ELISA for *L. dispar* hsp70 was performed according to general practice: samples were first incubated with monoclonal anti-Hsp70 mouse IgG1 (dilution 1:5000) (clone BRM-22, Sigma Aldrich, USA) for 12 h at 4 °C, and then for 2 h at 25 °C with secondary anti-mouse IgG1 (gamma-chain)-HRP conjugate (dilution 1:5000) antibodies (Sigma Aldrich, USA). Chromogenic substrate 3, 3’, 5, 5’-Tetramethylbenzidine (TMB) was used as a visualizing reagent. Absorption was measured on a microplate reader (LKB 5060-006, Austria) at 450 nm. To enable statistically valid comparisons of experimental groups across multiple microplates, each microplate contained serial dilutions of standard hsp70 (recombinant hsp70, 50 ng/mL), used for the hsp70 standard curve, and homogenized brain tissues pulled by each treatment that were loaded on the microplates in a matched design, ensuring that each data point represented the mean of three replicates from each experimental group.

Western blots were used to detect the presence of heat-shock protein 70 isoforms. Brain tissue homogenates were separated by SDS PAGE electrophoresis on 12% gels, according to Laemmli^50^. Protein transfer from the gel to the nitrocellulose membrane (Amersham Prothron, Premium 0.45 mm NC, GE Healthcare Life Sciences, UK) was left overnight at 40 V and 4 °C. Monoclonal anti-hsp70 mouse IgG1 (1:5000 dilution, clone BRM-22, Sigma Aldrich) and secondary mouse anti-mouse Hsp70 horseradish peroxidase conjugate antiserum (1:10,000 dilution, Sigma-Aldrich) were used for detection of hsp70 expression patterns in *L. dispar* larval brain tissue. Bands were visualized using chemiluminescence (ECL kit, Amersham).

This study identified the hsp70 concentration in brain tissue and specific activities of total ACP and ALP in the larval midgut as the most promising biomarkers, which are sensitive and have consistent responses to thermal stress. These three biomarkers were combined into an IBR analysis according to Beliaeff and Burgeot^51^. The value of each biomarker (Xi) was standardized by the formula Y_i_ = (Xi − mean)/SD, where Yi is the standardized biomarker response, and mean and SD were obtained from all values of the selected parameters. The next step was describing Z_i_ as Z_i_ = Y_i_ or Z_i_ = − Y_i_, depending on whether the temperature treatment caused induction or inhibition of the selected biomarkers. After finding the minimum value of Z_i_ for each biomarker (min), the scores (S_i_) were computed as S_i_ = Z_i_ + |min|. Scores for biomarkers were used as the radius coordinates of the studied biomarker in the star plots. Star plot areas for the three-biomarker assembly, positioned in successive clockwise order—Hsp70, total ACP, and ALP, were obtained from the following formulas: ${A}_{i}=\frac{{S}_{i}}{2*\mathrm{sin}\beta }\left({S}_{i}*\mathrm{cos}\beta + {S}_{i+1}*\mathrm{sin}\beta \right)$, $\beta = {\mathrm{tan}}^{-1}\left(\frac{{S}_{i+1}*\mathrm{sin}\alpha }{{S}_{i}-{S}_{i+1}*\mathrm{cos}\alpha }\right)$,$\alpha =2\pi /n$ radians (*n* is the number of biomarkers). The IBR values were calculated as follows:$IBR= \sum_{i=1}^{n}{A}_{i}$, where A_i_ is the area represented by two consecutive biomarkers on the star plot. Excel software (Microsoft, USA) was used to calculate IBR values and generate star plots.

Statistical analyses were conducted in GraphPad Prism 6 (GraphPad Software, Inc., USA). Mean values ± standard errors of mean values (SEM) were calculated for the activity of enzymes, larval midgut mass, and the hsp70 concentration in brain tissue. D’Agostino-Pearson omnibus and Shapiro–Wilk tests were used to check the normality of data distribution. The effects of thermal treatments and their interaction on the variance of analyzed biomarkers in larvae from the polluted and the unpolluted forest were tested using two-way ANOVA with thermal treatments as fixed factors. For all comparisons, the level of significance was set at *p* < 0.05.

### Ethics approval

All animal procedures were in compliance with Directive 2010/63/EU on the protection of animals used for experimental and other scientific purposes and were approved by the Ethical Committee for the Use of Laboratory Animals of the Institute for Biological Research “Siniša Stanković,” National Institute of the Republic of Serbia, University of Belgrade.

## Supplementary Information

Supplementary Information.

## Data Availability

The datasets used and/or analyzed during the current study are available in the *The effects of temperature stress and population origin on the thermal sensitivity of Lymantria dispar L. (Lepidoptera: Erebidae) larvae* repository, https://data.mendeley.com/drafts/5m6sjsdrg8.
