# Supplementary material for: The effects of temperature stress and population origin on the thermal sensitivity of Lymantria dispar L. (Lepidoptera: Erebidae) larvae
Source: Sci Rep. 2022 Dec 17;12:21858. doi: 10.1038/s41598-022-26506-2 (PMC9759568; doi:10.1038/s41598-022-26506-2)
Supplement: Supplementary file 1 — Supplementary Information. [file 41598_2022_26506_MOESM1_ESM.pdf]

Figure 1 - NATIVE PAGE gel stained for alkaline phosphatase in the midgut of 5th instar *Lymantria dispar* larvae exposed to different temperature treatments from unpolluted (UP) and polluted (PP) forest with enzyme isoforms. Gels were incubated with specific substrate for 2 hours.

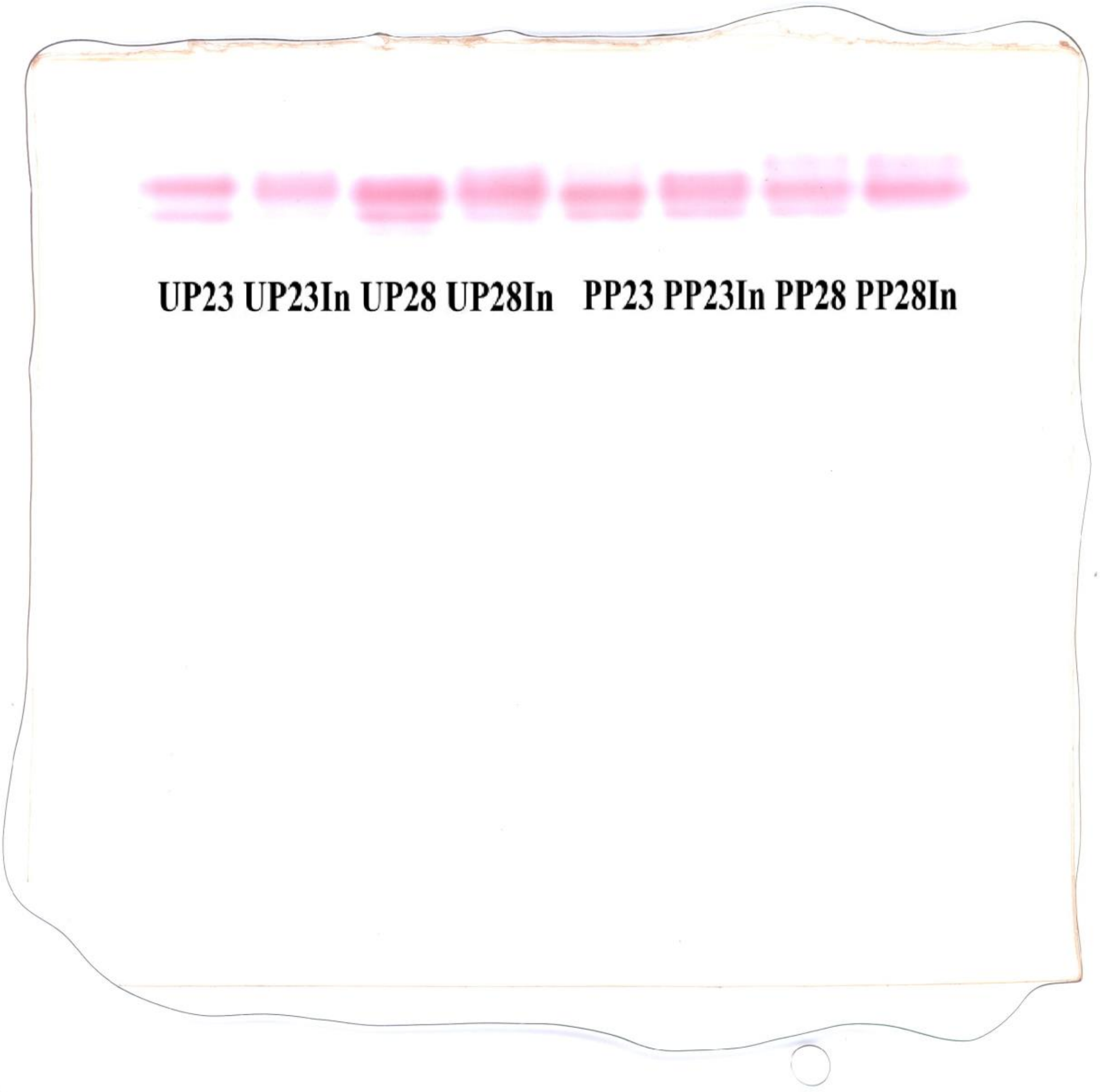

Figure 2 - NATIVE PAGE gel stained for alkaline phosphatase in the midgut of 5th instar *Lymantria dispar* larvae exposed to different temperature treatments from unpolluted (UP) and polluted (PP) forest with enzyme isoforms. Gels were incubated with specific substrate for 10 hours.

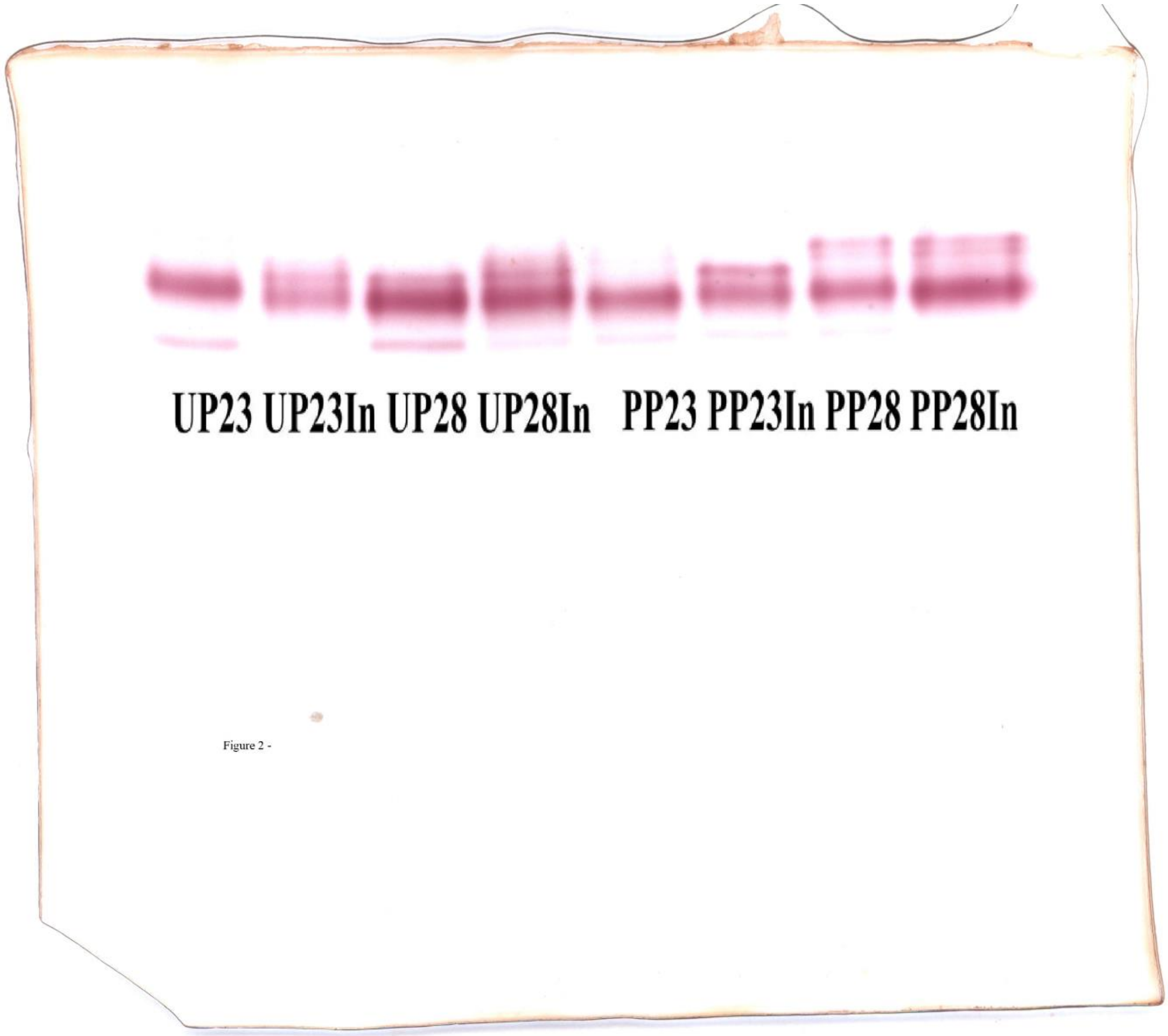

Figure 3 - NATIVE PAGE gel stained for total acid phosphatase in the midgut of 5th instar *Lymantria dispar* larvae exposed to different temperature treatments from unpolluted (UP) and polluted (PP) forest with enzyme isoforms.

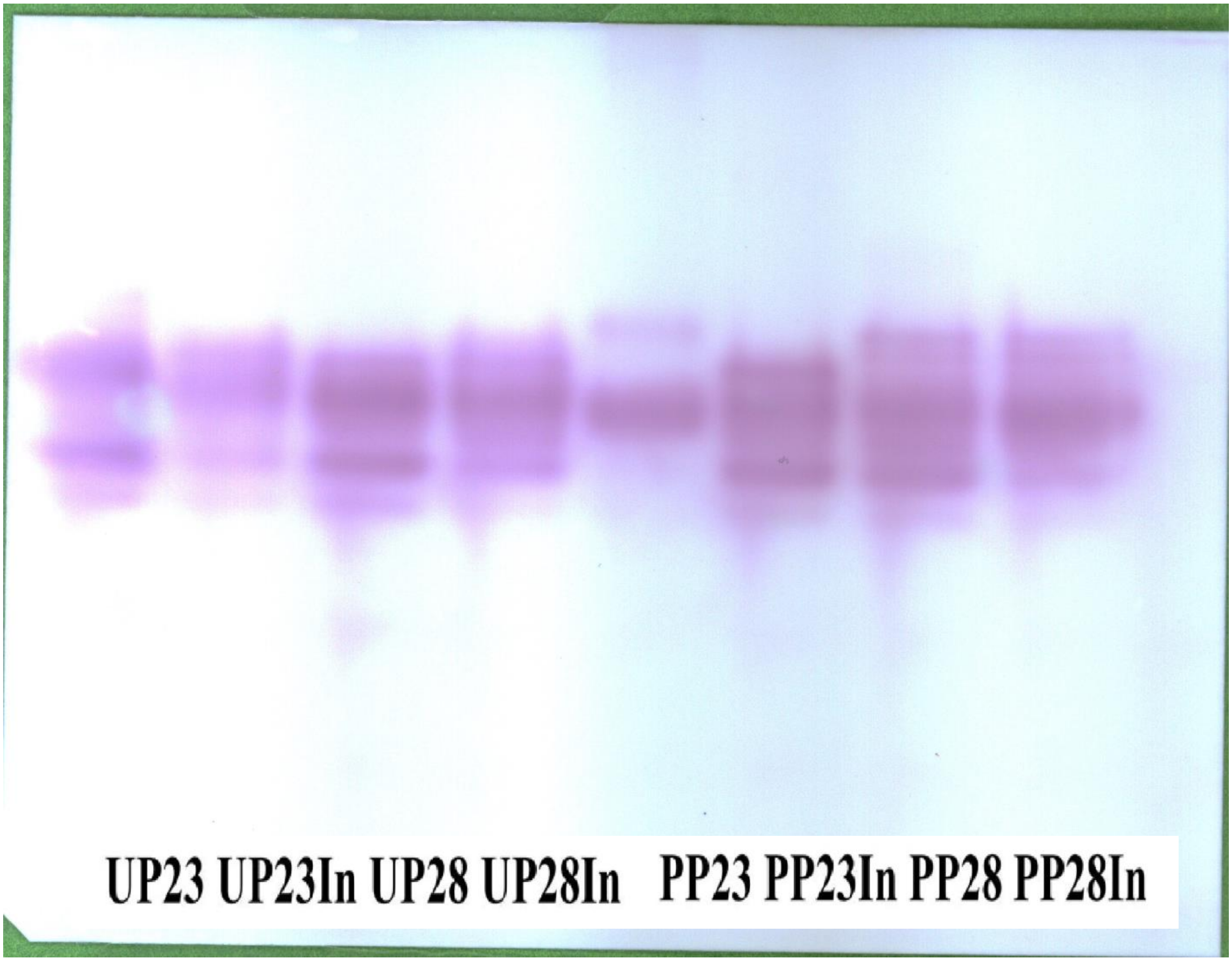

Figure 4 - Western blot of heat shock protein 70 (hsp70) from brain tissue of 5th instar *Lymantria dispar* larvae exposed to different temperature treatments from unpolluted (UP) and polluted (PP) forest

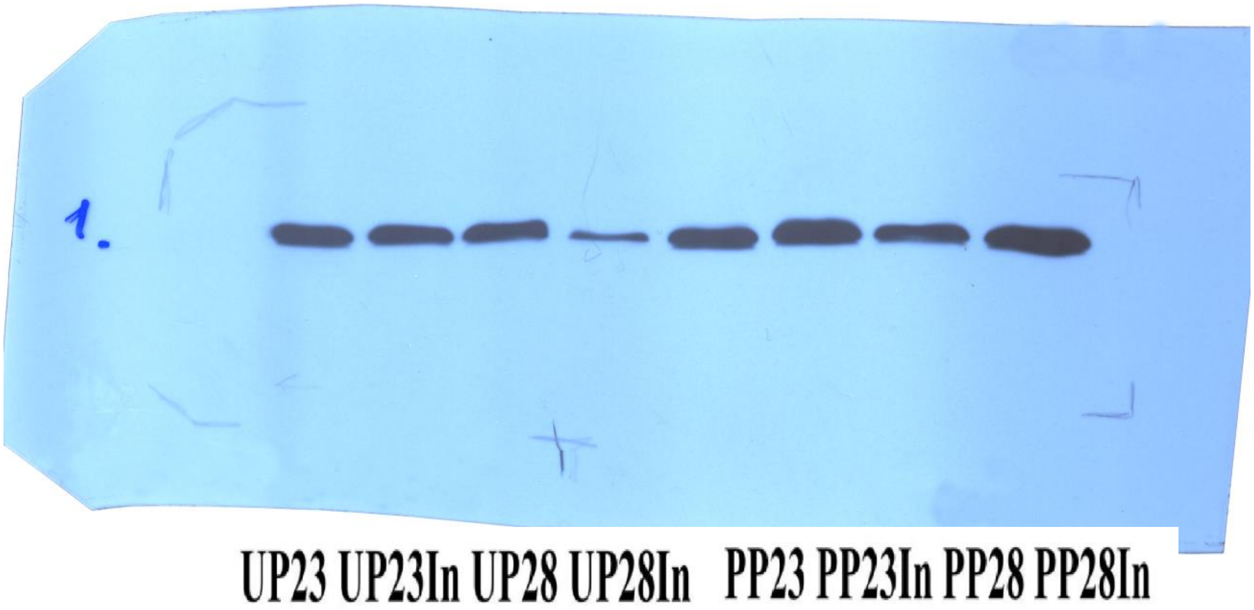

Figure 5 - Anti-actin internal control for hsp 70 Western blot. The difference in gels is the time of exposure (6 and 3 min, respectively). On the basis of actine control we have standardize hsp 70 Western blots in our experiment.

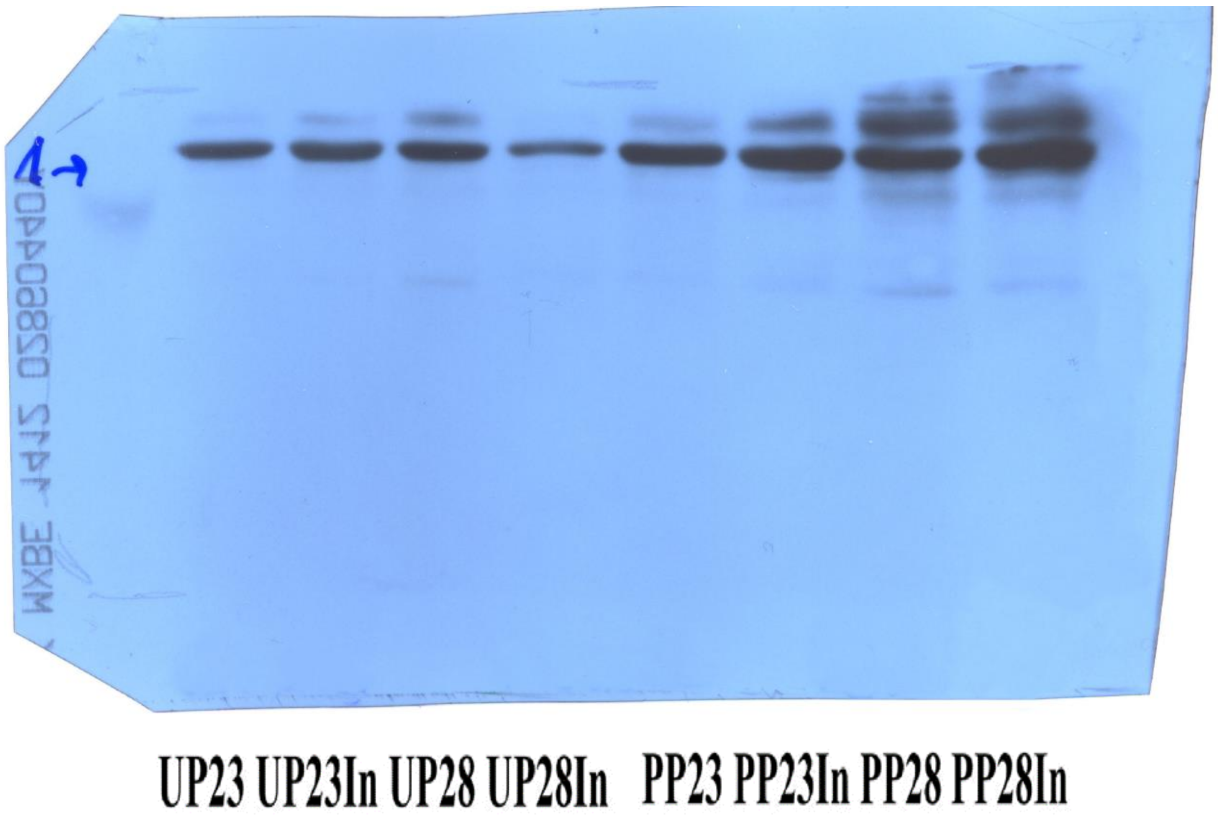

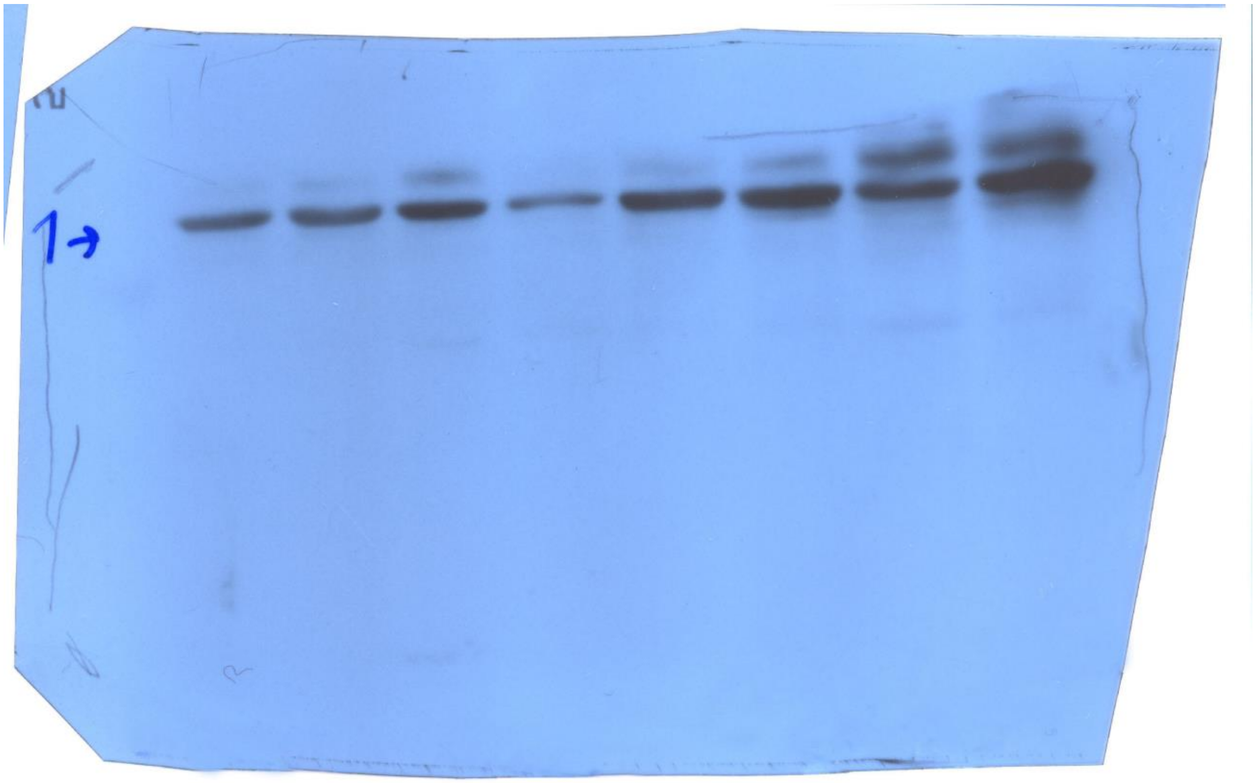

UP23 UP23In UP28 UP28In PP23 PP23In PP28 PP28In
